# Bibliometric analysis of trends and issues in traditional medicine for stroke research: 2004–2018

**DOI:** 10.1186/s12906-020-2832-x

**Published:** 2020-02-07

**Authors:** Lieyu Huang, Xuefeng Shi, Nan Zhang, Ya Gao, Qian Bai, Liming Liu, Ling Zuo, Baolin Hong

**Affiliations:** 1 School of Management, Beijing University of Chinese Medicine, No.11 North 3rd Ring Road East, Chaoyang District, Beijing, 100029 China; 2 National Institute of Chinese Medicine Development and Strategy (NICMDS), Beijing University of Chinese Medicine, No.11 North 3rd Ring Road East, Chaoyang District, Beijing, 100029 China; 3 Manchester Institute for Collaborative Research on Ageing (MICRA), Cathie Marsh Institute (CMI), Humanities Bridgeford Street (HBS) Building, the University of Manchester, Oxford Road, Manchester, M13 9PL UK; 4 School of Traditional Chinese Medicine, Beijing University of Chinese Medicine, Beijing, 100029 China

**Keywords:** Stroke, Traditional medicine, Bibliometric analysis, Trends, Web of science

## Abstract

**Background:**

Stroke is a major cause of death and disability worldwide. Over the years, traditional medicines for stroke treatment have undergone tremendous progress, but few bibliometric studies have been performed. This study explored the trends and issues relating to the application of traditional medicine in stroke research.

**Methods:**

A bibliometric search was performed in the Web of Science Core Collection database to identify studies that investigated the application of traditional medicine in stroke management. CiteSpace VI and Excel 2016 were used to analyze information from the retrieved studies. Activity index and attractive index were used to explore the worldwide development modes.

**Results:**

A total of 1083 English articles published between 2004 and 2018 were identified. Over the last 15 years, the developments in research occurred in three geographic clusters. The development modes were investigated and classified into 4 categories. In mainland China, the number and impact of research showed an increasing trend over the study period. The United States played a leading role in this topic. Three clusters of institutes and the majority of authors mainly came from South Korea, Taiwan and mainland China. Reperfusion injury and angiogenesis were identified as the potential topics likely to dominate future research in this field.

**Conclusion:**

The progress of studies on traditional medicine for stroke could be explained by the global attention to traditional medicine, the geospatial proximity for research collabration, and the increasing resources invested. Based on a large amount of existing research, researchers engaged in this topic should objectively consider the influential studies to identify and solve the common issues worldwide.

## Background

Stroke, consisting of ischemic stroke and hemorrhagic stroke, is increasingly being recognized as a serious worldwide public health issue [1]. In 2017, there were approximately 104.2 million stroke patients and 11.9 million new cases of stroke globally [2]. A number of cross-sectional studies relating to stroke have reported that stroke is the second leading cause of death [3] and the third leading cause of disability-adjusted life years worldwide [4, 5]. Stroke not only affects people’s health and the quality of life in the course of disease, but also requires substantial health care resources including medical costs and long-term care costs [6], which imposes a significant economic burden on families and healthcare systems. In the United States (USA) only, the total annual costs of stroke-related expenditures are estimated to increase by 129% each year and the costs are expected to reach US$240.67 billion by 2030 [7]. Recent evidence suggests that both low- and middle-income countries and high-income countries should redirect more resources to stroke research [8]. Currently, stroke treatment has been challenging due to lack of effective management strategies and interventions. According to World Health Organization (WHO), traditional medicine, complementary medicine and alternative medicine (TCAM) is defined as the sum total of knowledge, skill and practices in different cultures applied in the process of prevention, diagnosis and treatment [9], particularly having diverse health benefits for chronic and non-communicable diseases, including stroke [10]. Some investigations concerning the use of traditional Chinese medicine (TCM) in stroke patients based on a large cohort study have revealed that patients with stroke had a higher usage of TCM than those without stroke [11, 12]. In Korea, a national study published a handbook and white paper covering 9 years of clinical application of traditional Korean medicine for stroke patients [13]. Previous studies also recognized the currently and potentially significant role played by TCAM in the promotion of modern biomedicine in western countries [14]. These indicate the growing use of TCAM in stroke treatment and research. For TCAM as a whole, it consists of two parts: one is traditional medicine deeply rooted in the country culture and the other one is complementary medicine or alternative medicine as a set of health care practices neither part of the country’s own traditions or conventional medicine nor accepted into the mainstream health-care system of that country. Due to the interchangeable use of these terms in different countries, we used TCAM in this article referring to all traditional medicine, complementary medicine and alternative medicine, except where the use of terms “complementary” or “alternative” was necessary in some contexts.

Given the growing population with stroke and the current understanding of TCAM for stroke, an integrated and holistic analysis of trends and issues on this topic is required to provide new perspectives for future research. To our knowledge, although bibliometric methods have been applied in many research fields, including TCAM [15], no detailed bibliometric analysis has been performed on the topic of TCAM for stroke. The purpose of this study was to analyze the prevailing situation of stroke research using TCAM at a global scale, highlight the trends in several key countries, explore changes in journals and the networks among institutes and authors, and finally summarize the key areas on the topic. Therefore, we comprehensively employ several bibliometric approaches based on bibliographic records on this topic to achieve the goal stated above.

## Methods

### Literature search

One of the most well-known databases for conducting bibliometric analysis is the Web of Science Core Collection database (WoSCC), currently covering 18,000 journals which classifies information into 256 subject categories. The Citation Index applied in WoSCC is standardized to ensure the comprehensiveness of bibliographic records of all related influential and high-quality articles. Therefore, the relevant bibliographic records on the topic of TCAM for stroke were derived from WoSCC.

At the beginning of the study, all articles involving TCAM for stroke were searched in WoSCC. However, the number of articles before 2004 was relatively small and did not display a stable trend, compared with the later period after 2004, which suggested that this research field is relatively young. Therefore, we focused on the period from 2004 onwards to identify a trend with obvious changes in line with the requirements of a bibliometric analysis.

Literature retrieval was performed by two authors independently on January 1, 2019 to obtain the initial data from WoSCC and to avoid the bias caused by daily database updates. The search strategy was as follows: (Topic = (stroke AND (traditional medicine OR complementary medicine OR alternative medicine))) AND LANGUAGE: (English) AND DOCUMENT TYPES: (Article OR Review). Timespan ranged from 2004 to 2018, 15 years in total. A total of 1083 records were collected from WoSCC. The organized data were imported into Excel 2016 and CiteSpace VI for further systematic analysis.

### Analysis methods

To analyze the geographical distribution and collaboration, a geographic file was generated by CiteSpace and imported into Google Fusion Table in order to overlay the heat map and collaboration network of the articles on the world map. The literature analysis report from WoSCC was prepared in Excel 2016 to calculate activity index (AI) and attractive index (AAI) for determining the dynamic developments in a country. AI indicates the relative research effort of a country to a research field based on publications and AAI characterizes the relative impact of a country on a research field based on citations [16]. To assess the distribution and development of journals, overlay map was directly generated by CiteSpace [17, 18]. In addition, the 10 journals with the highest number of articles on this topic in the last 15 years were visualized with the river chart. CiteSpace was used to explore collaboration networks among institutes and authors and to construct the visual maps, while the operating parameter of selection criteria in CiteSpace was set as the top 50 most cited articles each year. The keywords of bursts were also identified by CiteSpace.

## Results

### World distribution and collaboration

**Fig. 1:**
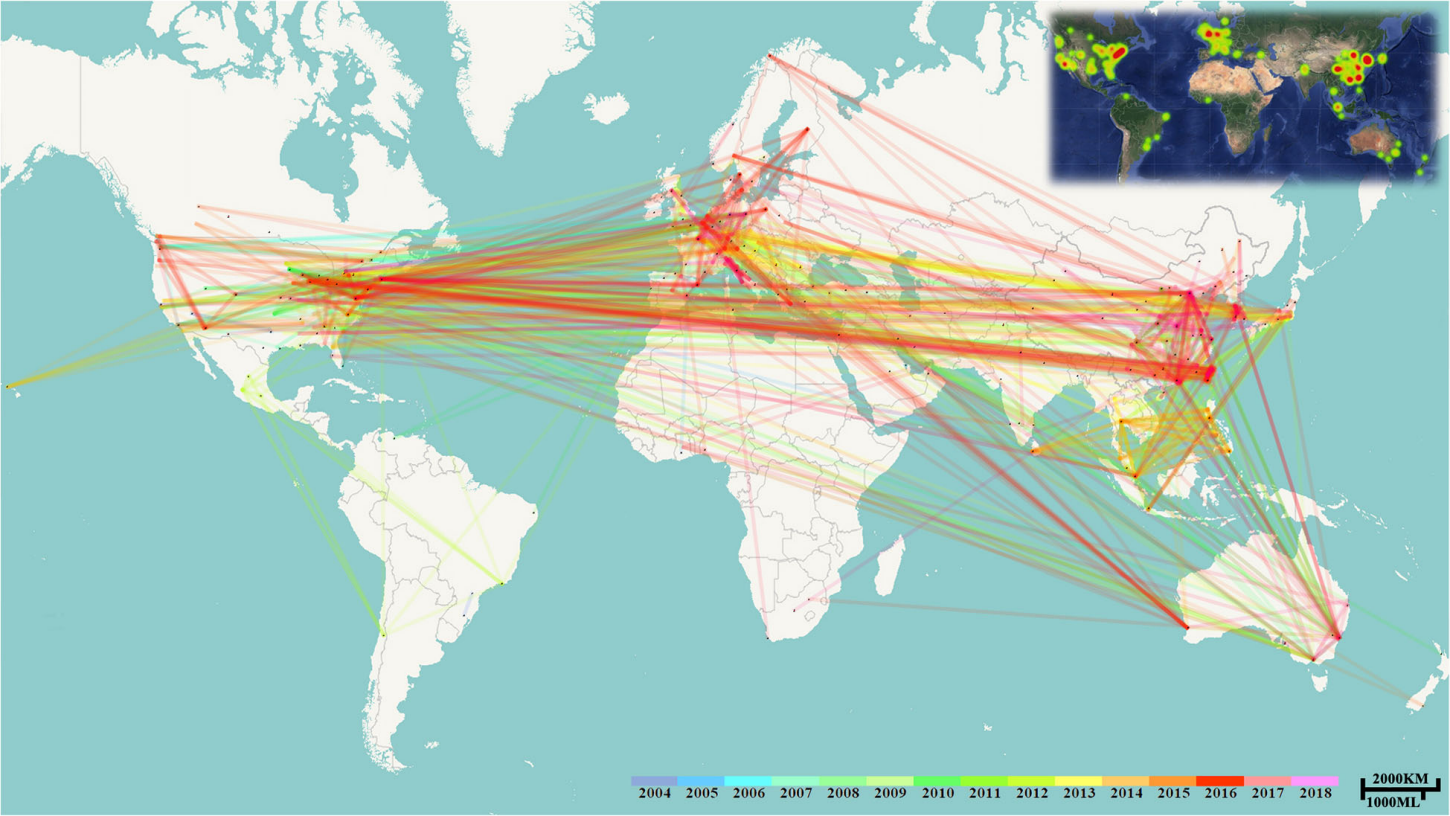
The world distribution and collaboration on the topic of TCAM for stroke. The distribution heat map was created using the Google Fusion Table and the collaboration map was created using CiteSpace VI software. Lines link the nodes on the map, representing the worldwide collaboration among institutes. The range and the color of the heat on the top-right corner indicate the number of articles at specific locations

Geographical data of articles was extracted by CiteSpace and imported into Google Earth and Fusion Table to create the collaboration network and the world distribution map (Fig. 1). The lines linking nodes on the map represent the worldwide collaboration among institutes. The overall collaboration network is displayed with different colors by year. A majority of collaboration networks were among USA, mainland China (hereinafter China), Taiwan, Australia, France, and Italy. The colors of lines were divided into warm colors and cold colors, broadly separating research into two periods, before or after 2012. The lines with warm colors were conspicuous and connected the key countries/regions (hereinafter countries), suggesting that worldwide collaboration began to increase after 2012. In contrast, the lines with cool colors mainly covered North America and Europe, which suggests that the initial research before 2012 concentrated in these two areas. The heat map of articles on the top-right corner reveals the aggregation and centrality of research worldwide. The range and the color of the heat stand for the number of articles at specific locations. It was apparent that there were three main research clusters, including east coast of North America, Northern Europe and East Asia. The other areas were fragmented. Specifically, the majority of articles came from developed countries, such as USA, the United Kingdom (UK) and South Korea, around which clusters covering these areas aggregated.

### Development of the top 5 countries

The number of articles increased from 18 in 2004 to 152 in 2018, with a compound annual growth rate of about 20.57% between 2010 and 2016. The top 5 countries, in terms of number of articles, accounted for over 80% of the total articles globally.

**Fig. 2:**
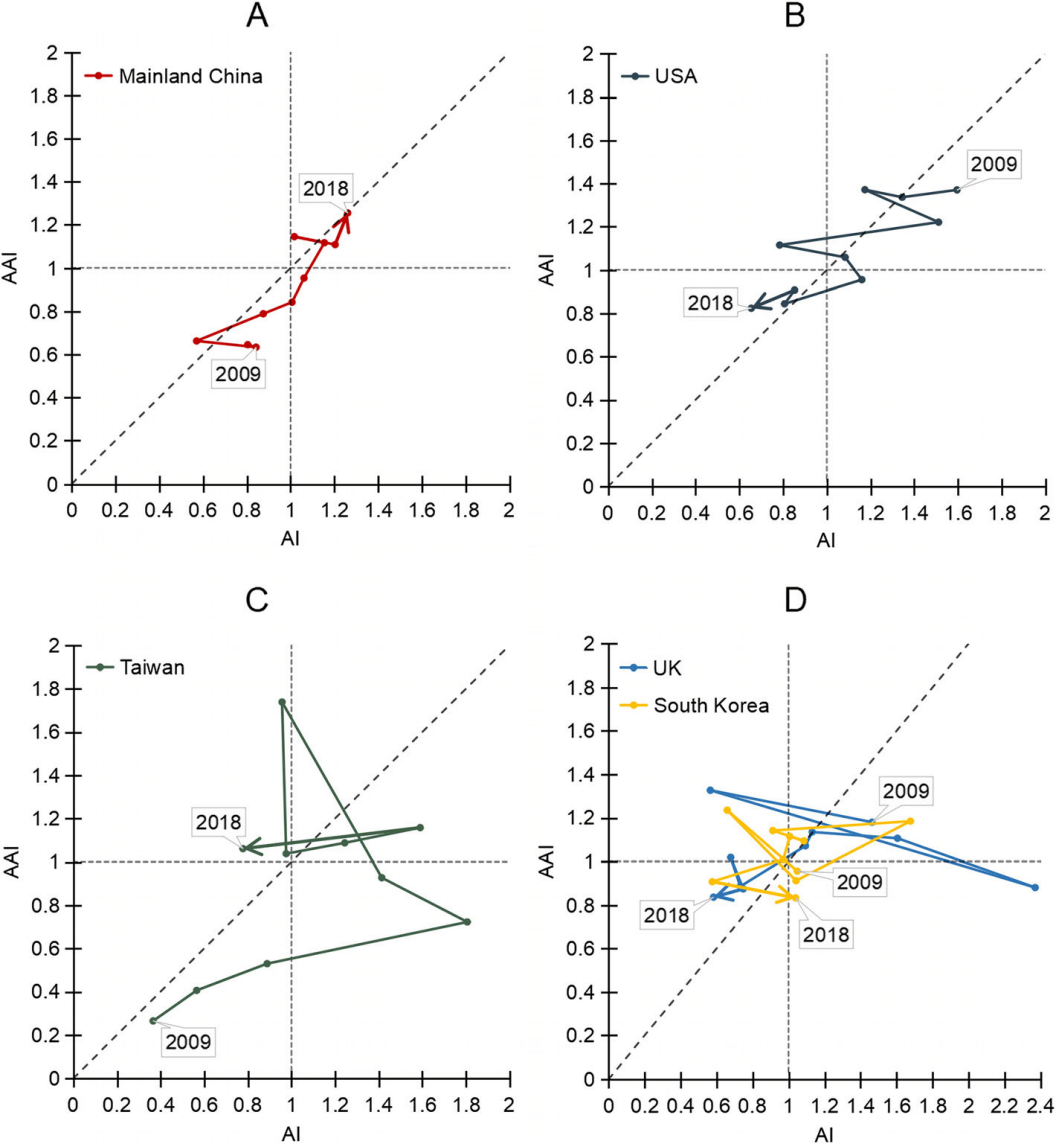
The relational chart of AI and AAI from 2009 to 2018 for the top 5 countries. The dotted line AI = 1 represents the global average contribution to TCAM for stroke, and AAI = 1 the global average impact. The reference line y = x represents the balance of effort and impact of a country’s research

AI = 1 and AAI = 1 represented global average levels of research power and influence, respectively [17]. If AI or AAI was greater than 1, then the research power or the academic influence of a country was higher than the global average, and vice versa. From the perspective of development in the last 10 years, the top 5 countries were grouped into four categories according to two characteristics (Fig. 2; Additional file 1). The first category comprised countries for which the articles number and influence showed an increasing trend compared with the global average, including China and Taiwan. The second category comprised countries for which the articles number and influence showed a decreasing trend compared with the global average such as USA, South Korea and UK. In addition, based on the degree of deviating from the diagonal line, the top 5 countries were further divided into two types, China and USA being the balanced type, while South Korea, Taiwan and UK being the unbalanced type. Balance type referred to countries with changes falling roughly along the reference line y = x, where x was AI value and y was AAI value. Therefore, research level in China increased significantly since 2009 with overall balance between the quantity and the influence, both which were slightly higher than the global average in the last 4 years. Notably, results from China were somewhat biased by AI, suggesting that the growth of China was largely driven by the number of articles rather than the influence of articles. In spite of the downward trend, USA still dominated on this topic with many high-quality studies ranking above the global average. The fluctuations in Taiwan, South Korea and UK may be ascribed to the small number of articles. Nevertheless, compared to the global average level, South Korea and UK produced articles with a relatively higher quality, and the impact of articles from Taiwan generally grew.

### Distribution and development of journals

**Fig. 3:**
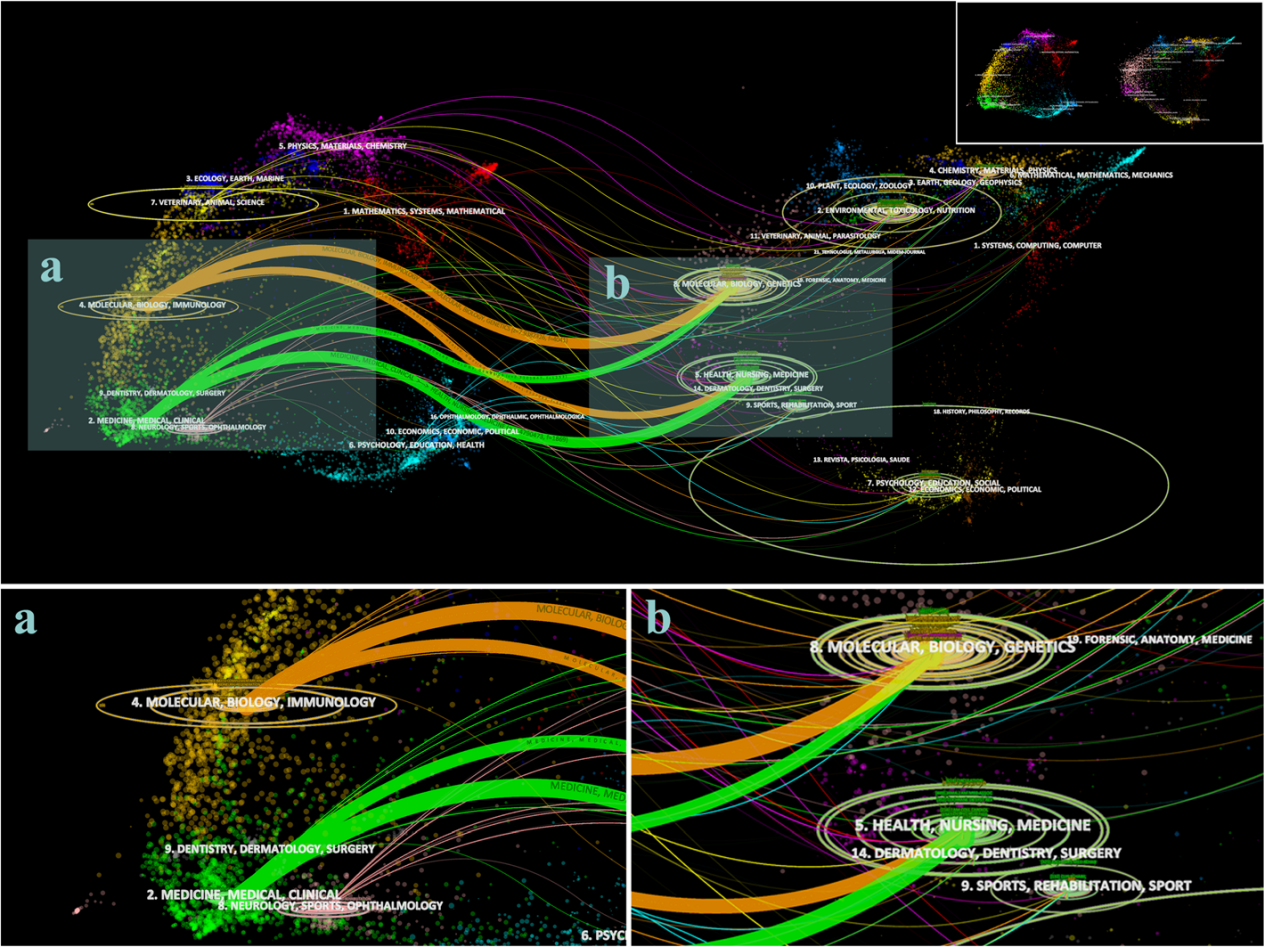
Dual-map overlay of journals related to TCAM for stroke topic. A total of four citation paths were identified. The journals of citing articles are on the left (**a**), and the journals of references on the right (**b**)

The Dual-map overlay of CiteSpace shown in Fig. 3 was utilized to analyze the relationship between citing articles and cited articles, to reveal the major disciplines associated with TCAM for stroke. The thumbnail on the top-right corner shows the original map before adding overlay. Each journal is presented a circle and different colors of circles indicated different disciplines of journals. The journals of the citing articles are shown on the left, and the journals of the referenced articles are shown on the right. The left part presents the application fields, and the right part shows the research foundations. In the main body of Fig. 3, the lines linking different circles stand for citations and the width of lines implies the strength of connection between two main disciplines. The height of ovals refers to the number of articles and the width denotes the number of authors. Four lines (two yellow and two green) were identified. The main citing articles belonged to two disciplines: Biology and Immunology, and Medical and Clinical. The citation pathways of these disciplines were heading to Biology and Genetics, and Health and Medicine. The enlargement of the two main areas was on the bottom.

**Fig. 4:**
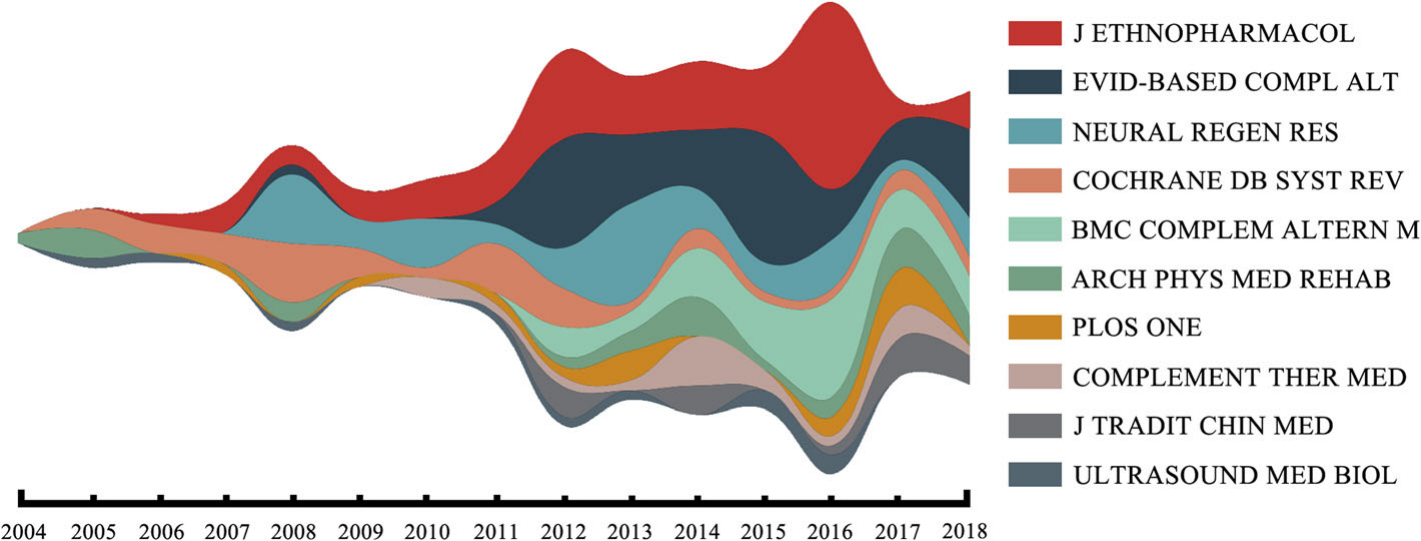
The river chart of the 10 journals with the highest number of articles. Each journal has its theme color and the width of lines represents the number of articles. The flow direction is from left to right, ranging from 2004 to 2018

The river chart of the 10 journals with the highest number of articles on this topic is shown in Fig. 4. The results show that some journals persistently kept tracking this research field, such as Journal of Ethnopharmacology. Figure 4 also shows that the number of articles increased from 2012, after which several other journals started to focus on this topic. The average impact factor (by 2018) of the 10 journals was about 2.77.

### Collaboration of institutes and authors

**Fig. 5:**
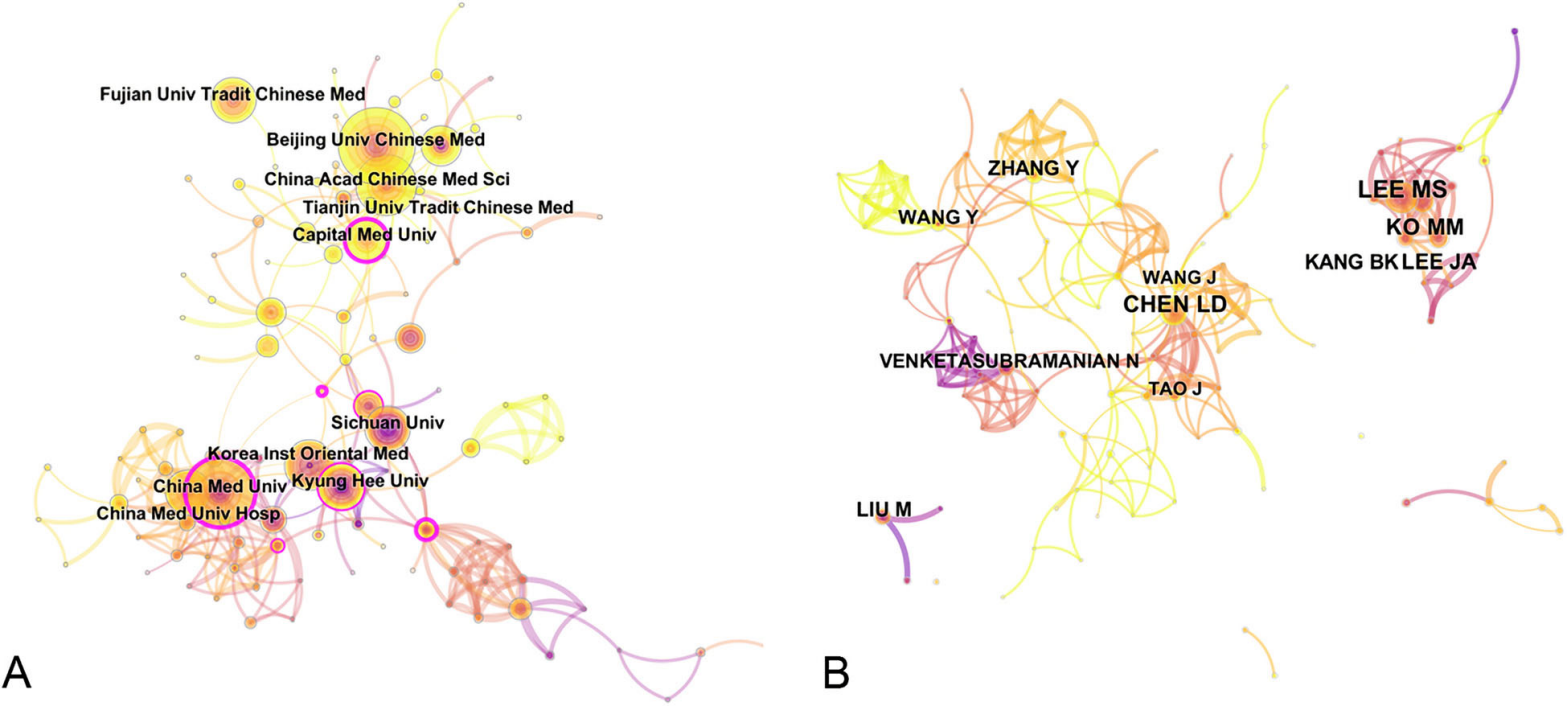
Collaboration among institutes (**a**) and authors (**b**). Different nodes represent elements such as an institute or an author, and links between nodes represent relationships among collaborators. The color of nodes and lines stand for different years: the lighter the color, the closer the time. The outermost purple rounds of circles denote the centrality level. Nodes with high centrality are regarded as turning points or pivotal points in the research field

In this part, CiteSpace was applied to generate the collaboration network of institutes and authors, as shown in Fig. 5. For institutes, the size of nodes represents the number of articles per an institute. The lines represent the collaborative relationships among institutes. The colors of circles and links represent different time periods. In total, 156 institutes contributed to this topic. Three clusters were easily distinguishable, where the collaborations were also evident. The first cluster comprised institutes from China, with Beijing University of Chinese Medicine and Capital Medicine University being the main ones. The second cluster comprised China Medical University and its affiliated hospital from Taiwan and the third cluster comprised South Korean institutes. In accordance to the color of nodes and lines, the cluster from China was relatively younger than the other two clusters despite having a large number of articles. Two clusters had deep purple round circles, which implied that the turning points tended to exist in clusters with longer history.

Over 300 authors contributed to the research field. Figure 5 (B) shows the collaboration network among authors. Most of the authors were from China and South Korea. Similar to institutes, authors from South Korea began research in the field earlier than those from China and Taiwan. The difference with institutes is lack of obvious circles with purple round emerging. In other words, as the explanation of circles with purple round or turning points mentioned above, only few authors had a great academic impact on this topic, which may be attributed to the short research period these authors were engaged in this topic.

### Keywords of bursts

**Fig. 6:**
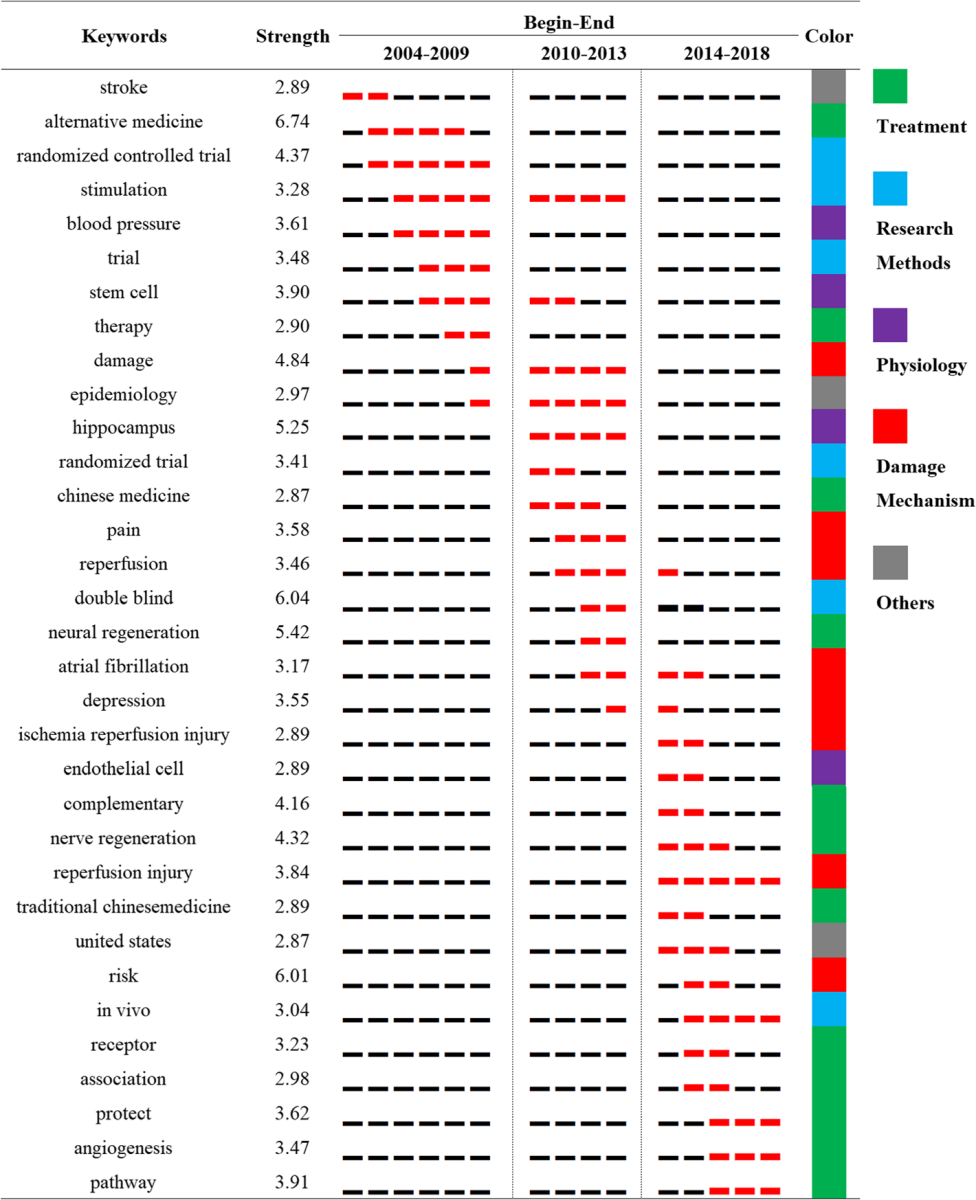
33 keywords with strong citation bursts in articles published. The strength value reflects the frequency of citation

Burst keywords are words cited frequently over a period of time. CiteSpace was used to capture burst keywords, the indicators of frontier topics over time. A total of 33 bursts keywords were obtained, together with their strength and the duration, as shown in Fig. 6. The value of strength of the burst keywords is indicated by the frequency of citation. Alternative medicine and risk had the highest strength value. The black line represents the period of study and the red line represents the period of citation bursts. According to the duration, the burst keywords were generally divided into three periods: from 2004 to 2009, from 2010 to 2013, and from 2014 to 2018. In terms of individual burst keywords, they were classified into five categories with different colors: treatment in green, research methods in blue, physiology in purple, damage mechanism in red, and others in gray.

## Discussion

### Data collection

To our knowledge, this is the first bibliometric analysis on TCAM for stroke based on WoSCC. Data obtained from WoSCC is visualized by CiteSpace tool to perform a high-quality bibliometric analysis of research development in a specific field for the Web of Science was the designed standard of CiteSpace. This study obtained original and review articles published between 2004 and 2018, relating to the continuously developing field of TCAM for stroke. All articles retrieved from the WoSCC platform were written in English. The interdisciplinary nature of research might induce the rapidly growing body of the relevant studies [19]. For this study, WoSCC had 1083 records between 2004 and 2018 based on a topic search of the term “stroke” and “TCAM” in titles, abstracts or indexing terms. If we regarded articles that cited at least one of the 1083 records as the relevant articles, the number could be as 15 times higher and too many records of low correlation were included. Therefore, we limited the search strategies to acquire a total of 1083 direct-related records on this topic for analysis. Data was verified and then synthetically analyzed by various bibliometric methods and software to receive relatively objective results. Although the WoSCC is continually updated, the newly added data in a short period may have limited influence on the final results of this study.

### World trend and development of major countries

Globally, there was an increase in research and collaborations in the last 5 years. Analysis showed that three centers, North America, Europe, and East Asia were involved in TCAM and stroke research. According to the Global Burden of Disease 2016 Study, Eastern Europe and East Asia had the higher age-standardized prevalence rates of stroke and countries in Eastern Europe, East Asia, and parts of Southeast Asia, Central Asia, and sub-Saharan Africa had the highest rates of stroke mortality [20]. However, these prevalence and mortality distributions did not geographically match the three research centers mentioned above. Thus, the presence of the three centers might be explained from the development of the research on TCAM. Following the initial efforts by the WHO to promote the global application of TCAM, the WHO Traditional Medicine Strategy 2014–2023 reported that, in addition to policy support, some global TCAM professional organizations have directed resources towards increasing awareness and research in TCAM through education and funding, especially in America, China and other countries in Europe [10]. This meant that TCAM strategies by the WHO also promoted scientific progress in TCAM field. As a consequence, numerous studies and articles on this topic were identified in the study period, especially a combination of stroke and other global issues. Another possible reason that may explain the three clusters is the geospatial proximity, an emphasis of spatial bibliometric analysis [21]. It was among geographically close regions that there were more collaborations and innovation processes [22].

We further determined the top 5 countries with the highest number of articles. We found that there was an overall increase in the research output. However, there were significant changes in global research landscape. Chinese researchers engaged in this topic accounted for over half of all countries, while the contribution from other countries showed an unsteadily increasing trend. That is to say, China dominates quantitatively in this research field. The H-index, as an indicator for assessing the quality of scientific output, is commonly used to calculate both the productivity and citation impact per an author or group of authors belonging to an institute or country [23]. H-index value is based on a list of publications ranked in a descending order by the Times Cited count, calculated by the principle that h articles are cited at least h times [24]. H-index is an accurate reflection of the academic contribution and achievement and is applied in many bibliometric analyses [25]. However, H-index is not suitable for comparing interdisciplinary fields [26]. To assess the development mode of countries on this topic behind H-index, we introduced the AI and AAI from literature and referred to the practical application in another study [27]. Considering the lag between the publication time and the time the article is cited, we used last 10 years in this study for analysis. China was increasing with general balance in AI and AAI for the last 10 years, while that of USA decreased in the same period. Especially since 2014, the number and the influence of Chinese research on this topic have been above the global average level. The remarkable development in China may be explained by not only the number of articles mentioned above and the promotion of TCM background, but also the boost of increased funding. A recent study has shown that nearly 80% of SCI-E articles from China received science funding, and the funding in China is the highest globally [28].

### Institutes and authors

In total, 1083 articles were published within two areas of journals including Molecular, Biology and Immunology, and Medicine, Medical and Clinical. The references of the 1083 articles headed towards two areas of journals as well: Molecular, Biology and Genetics, and Health, Nursing and Medicine. For the 10 journals with the highest number of articles on this topic, the IF varied greatly from 0.857 to 6.754, with an average of 2.77. Research and collaborations in South Korea and Taiwan began earlier than those in China. The result of top 10 authors was similar to that of the institutes. However, it should be noted that the names of authors from China have same abbreviations hence may exaggerate the number of authors. For instance, the abbreviations of both Wang Yan and Wang Yang were Wang Y. However, most of institutes and authors were from China and South Korea. It was Longa et al., the most cited authors, that provided a reliable method of non-invasive small animal model for studying reversible regional ischemia in rats without craniectomy in 1989 [29], widely cited by the subsequent studies on this topic.

### Research frontiers

Keywords were chosen to reflect the main focus of the entire research, according to the established principles of searching. Therefore, the CiteSpace was used to identify the burst keywords, which could be seen as the research trace over the past and the potential research frontiers in the future.

Thirty-three burst keywords were classified into five categories with different colors listing on the right of Fig. 6. Except for the “others” categoryThe other four categories were arranged in the order that they first appeared in Fig. 6 and further explained as follows: (1) the treatments were belonged to one aspect from the TCAM for stroke topic. Due to the synonyms extensionComplementary and alternative medicine had been shown in the past research. TCAM was widely used to promote neural or nerve regenerationWhich are concepts of modern medicine. The emerging keywords of this category including protectAngiogenesis and pathwaySuggested more refined treatments and more precise targets and pathways. TCM as the only one specific TCAM in the past 15 years signified the contribution of TCM for stroke [30]. (2) research methods included trials (double blind and randomized controlled) and research molding (simulation and rat). The burst keywords of the methods became more professional and specific. (3) physiology keywords included stem cellHippocampus and endothelial cellWhich may suggest the investigation over stroke itself was relatively discontinuous. This may be explained by the interdisciplinary nature of this topic. (4) the damage mechanism started from the general wordDamage. After a substantial amount of time had lapsedThe research point concentrated on the reperfusion injury and the risk. OverallResearch has focused on precise treatmentTargeted therapy and damage mechanismsWhile the stress on methods was fading

### Limitations and future perspectives

The exclusion of non-English articles, due to the unified language requirement of CiteSpace, may have resulted in incomplete coverage of articles published on this topic. However, given the global recognition of English, the influence of this exclusion on the results of this study is limited. The bibliometric analysis of this study did not consider the content of the articles.

By integrating the bibliography records on the topic, key elements were extracted from numerous articles, thereby providing high quality results. Given the evolution of fitness and medical models, TCAM has attracted global attention. The reasons behind the developments in each country require further investigation specifically focusing on stroke type (ischemic vs. hemorrhagic) and treatment (acute treatment vs. primary prevention vs. secondary prevention). Meanwhile, some countries should carry out research according to their own medical theory and tradition, such as the Ayurveda in India. Further studies using comprehensive search strategies are advocated to improve the interdisciplinary research on TCAM for stroke.

## Conclusion

This study provides a deeper insight into research on TCAM for stroke. Three cluster areas and three cluster institutes have been identified to be the ideal centers for collaboration. China has become the most productive country whilst USA still plays the leading role in TCAM research. Information regarding the main journals is presented for future research. Authors from Taiwan and South Korea held solid research foundation on this topic. Reperfusion injury and angiogenesis of targeting effect are likely to be the next frontier topics in this field.

## Supplementary information

**Additional file 1.** The AI and AAI dataset for Fig. 2. The detailed calculation process of AI and AAI for the top 5 countries on the topic of TCAM for stroke research.

## Abbreviations

AAI: Attractive index
AI: Activity index
TCAM: Traditional, complementary and alternative medicine
TCM: Traditional Chinese medicine
UK: The United Kingdom
USA: The United States
WHO: World Health Organization
WoSCC: Web of Science Core Collection database
